# Supplementary material for: Fatty acid desaturation and lipoxygenase pathways support trained immunity
Source: Nat Commun. 2023 Nov 15;14:7385. doi: 10.1038/s41467-023-43315-x (PMC10651900; doi:10.1038/s41467-023-43315-x)
Supplement: Supplementary file 1 — Supplementary Information [file 41467_2023_43315_MOESM1_ESM.pdf]

Supplementary Figures

Fatty acid desaturation and lipoxygenase pathways support trained immunity

Anaísa V. Ferreira[a,b*#], Juan Carlos Alarcon-Barrera[c#], Jorge Domínguez-Andrés[a], Ozlem Bulut[a], Gizem Kilic[a], Priya A. Debisarun[a], Rutger J. Röring[a], Hatice N. Özhan[a], Eva Terschlüsen[d] Athanasios Ziogas[a], Sarantos Kostidis[d], Yassene Mohammed[c], Vasiliki Matzaraki[a], George Renieris[e], Evangelos J Giamarellos-Bourboulis[e], Mihai G. Netea[a,f], Martin Giera[c*]

[a] Department of Internal Medicine and Radboud Center for Infectious Diseases (RCI), Radboud University Nijmegen Medical Center, 6500HB Nijmegen, The Netherlands
[b] Instituto de Ciências Biomédicas Abel Salazar (ICBAS), Universidade do Porto, 4050-313 Porto, Portugal
[c] Center for Proteomics and Metabolomics, Leiden University Medical Center, 2333ZA Leiden, the Netherlands
[d]Department of Medical Microbiology, Radboud University Medical Centre, 6500HB Nijmegen, The Netherlands
[e]4th Department of Internal Medicine, National and Kapodistrian University of Athens, Medical School, Athens, Greece.
[f]Department for Immunology and Metabolism, Life and Medical Sciences Institute (LIMES), University of Bonn, 53115 Bonn, Germany

#Equally contributing authors
*Corresponding authors:
AV Ferreira: anaisa.validoferreira@radboudumc.nl
M Giera: m.a.giera@lumc.nl

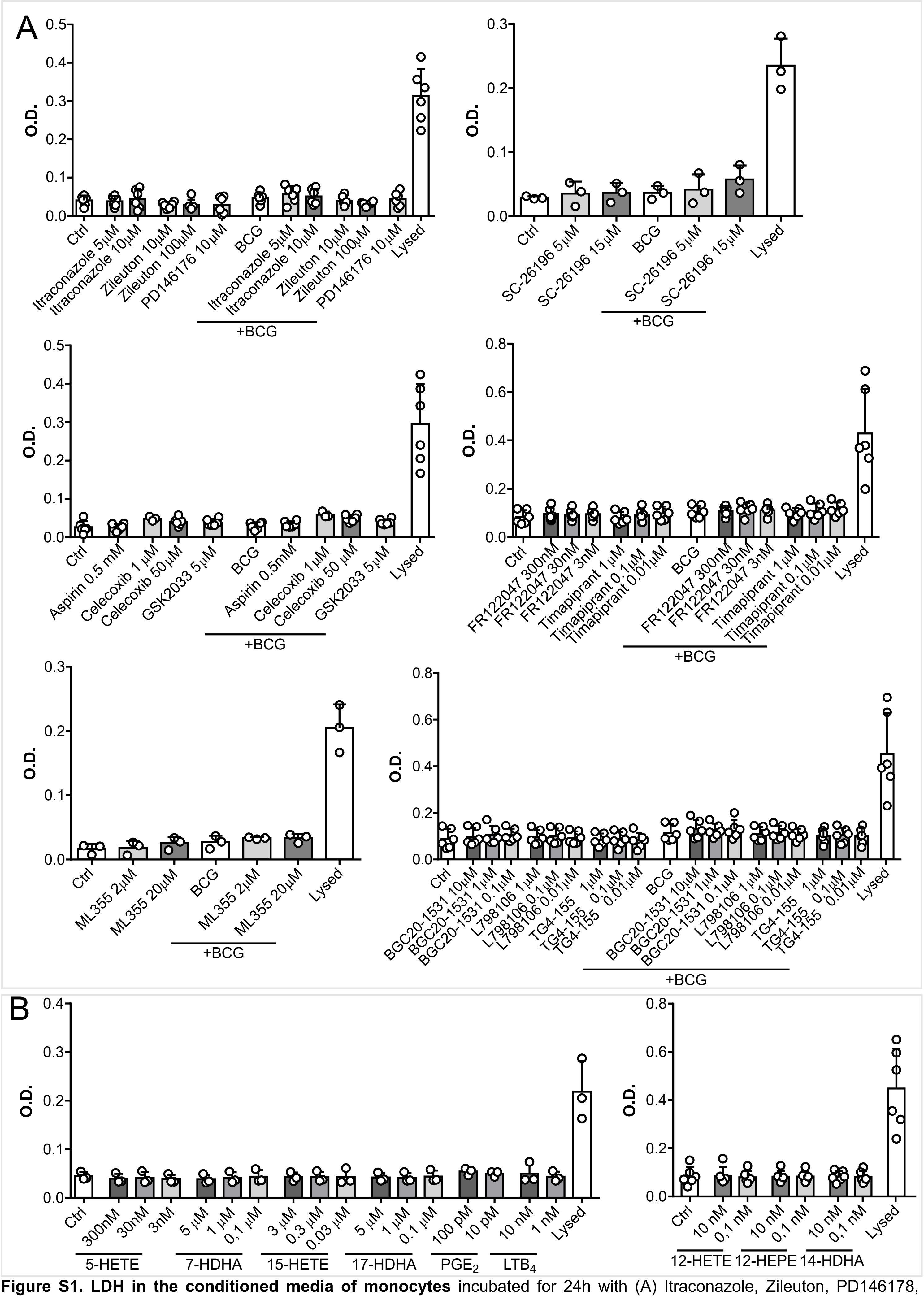

Figure S1. LDH in the conditioned media of monocytes incubated for 24h with (A) Itraconazole, Zileuton, PD146178, SC-26196, Aspirin, Celecoxib, GSK2033, FR122047, Timapiprant, ML355, BGC20-1531, L798106, TG4-155 alone or in combination with BCG (n=3/6 biological replicates pooled from 2 independent experiments, Mean+SD) and (B) 5-HETE, 7-HDHA, 15-HETE, 17-HDHA, LTB4, PGE2, 12-HETE, 12-HEPE, 14-HDHA (n=3/6 biological replicates, pooled from 1/2 independent experiments Mean+SD).

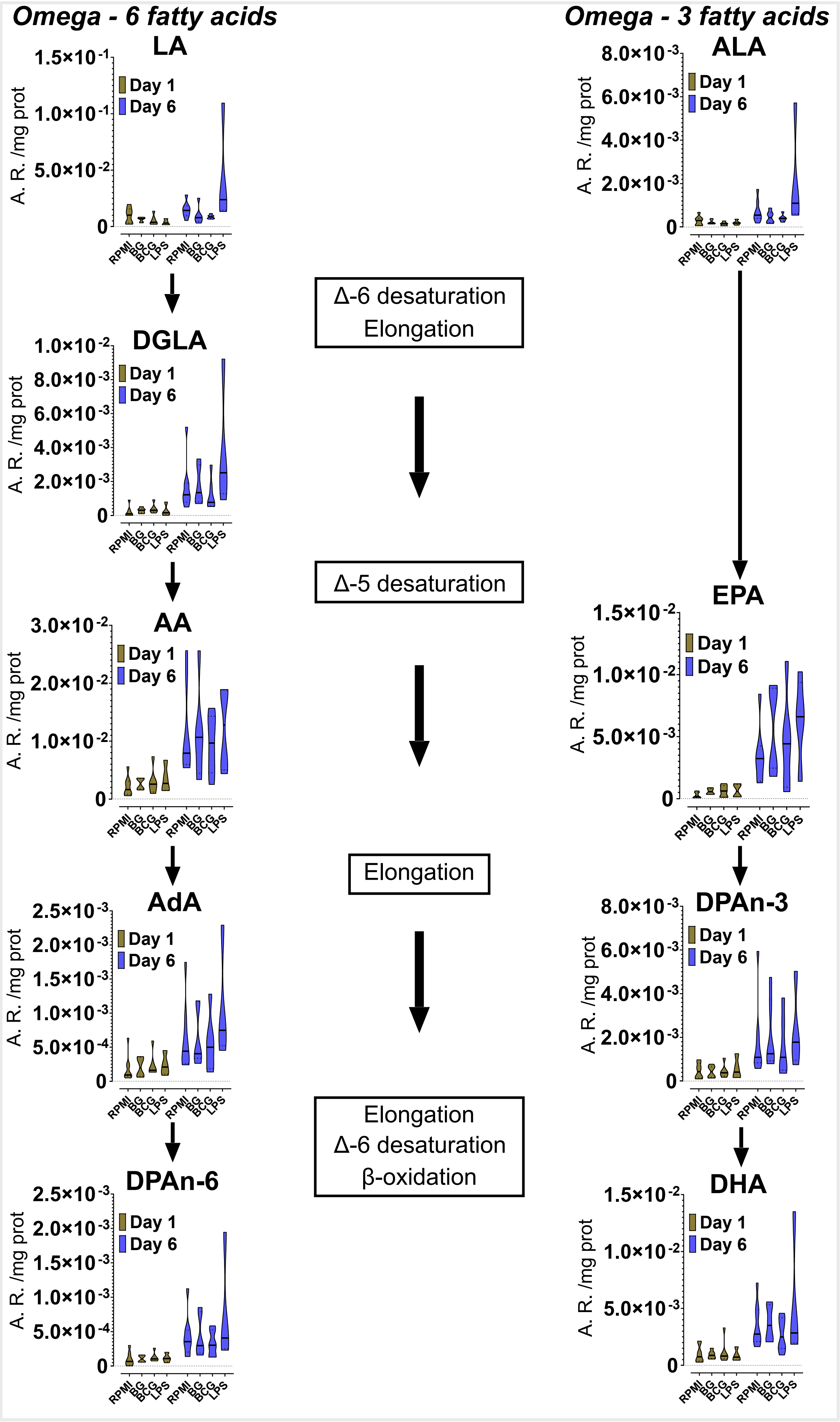

Figure S2. Area ratio (A.R) levels of PUFA corrected for internal standards and protein in the omega-6/3 pathways in stimulated monocytes at 24h with BG, BCG, and LPS and after 6 days of culture (n=4-8 biological replicates), Median and the interquartile region.

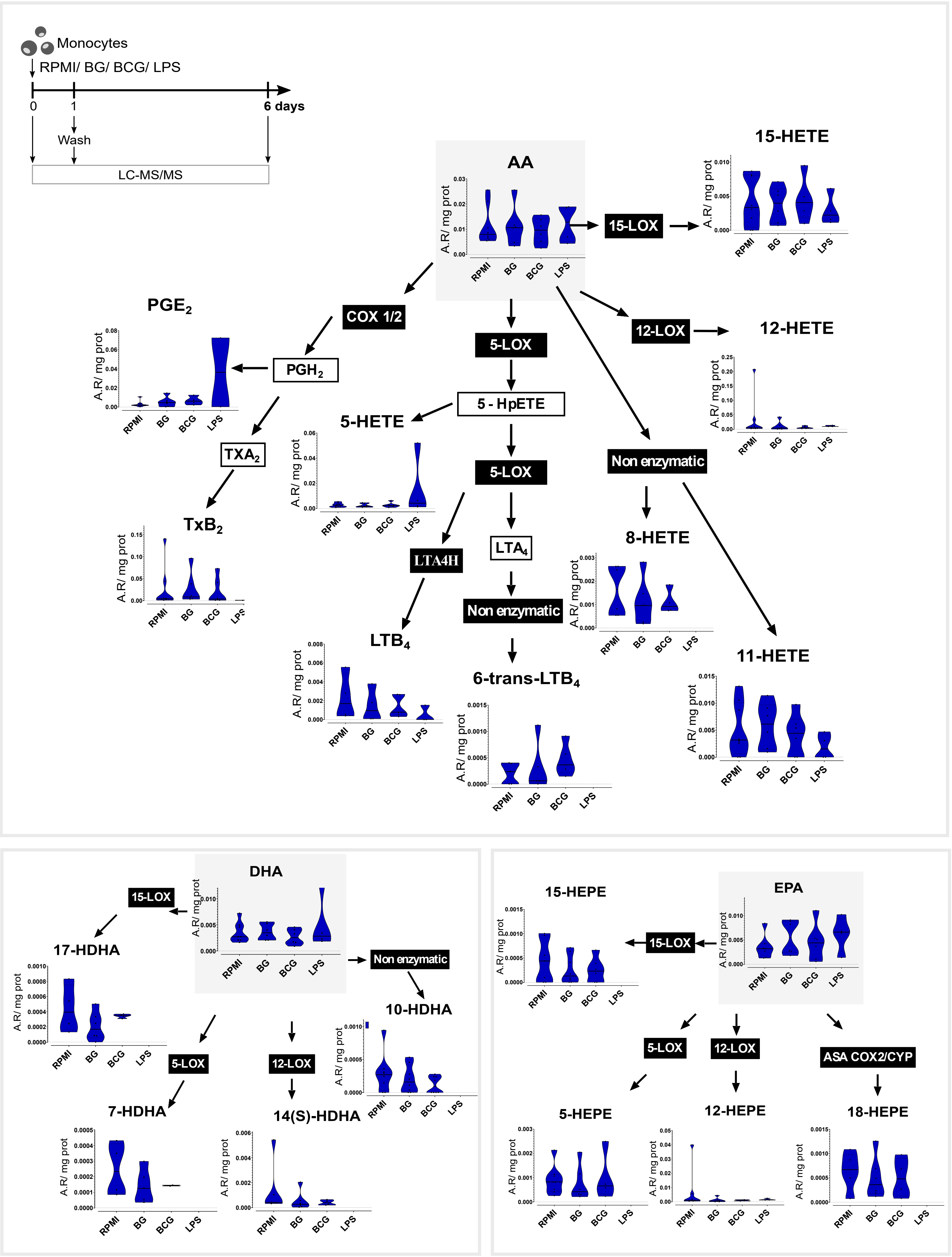

Figure S3. Area ratio (A.R) levels corrected to protein levels of PUFA and oxylipids of macrophages at day 6 of culture after in vitro exposure to BG, BCG or LPS (n=4-8 biological replicates), Median and the interquartile region. Data points below the technical limit of detection are not shown.

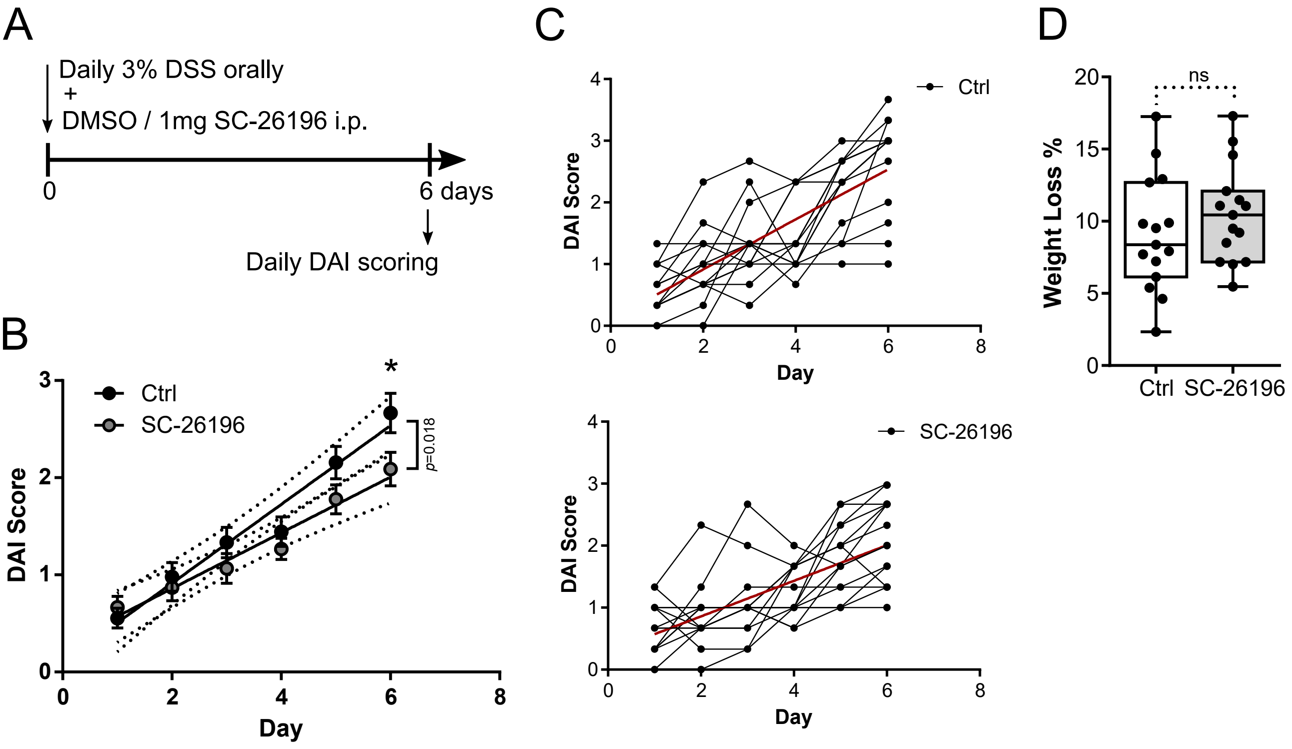

Figure S4. In vivo DSS-induced colitis DAI score and weight loss. (A) Schematic representation of the *in vivo* colitis model used. (B) DAI score (stool consistency, rectal bleeding, and weight loss) of mice with DSS-induced colitis and daily administration of 1 mg SC-26196 or vehicle control. (C) DAI score of individual mice in the vehicle control group (top panel) and in the SC-26196 group (bottom panel), linear regression is shown in red. (D) Weight loss of mice at day 6 (n=15 mice per condition pooled from 2 independent experiments), (A) Mean+SD, (D) Mean, 10-90 percentile and whiskers extend to the most extreme points. Mann-Whitney U test and linear regression analysis. ns: non-significant

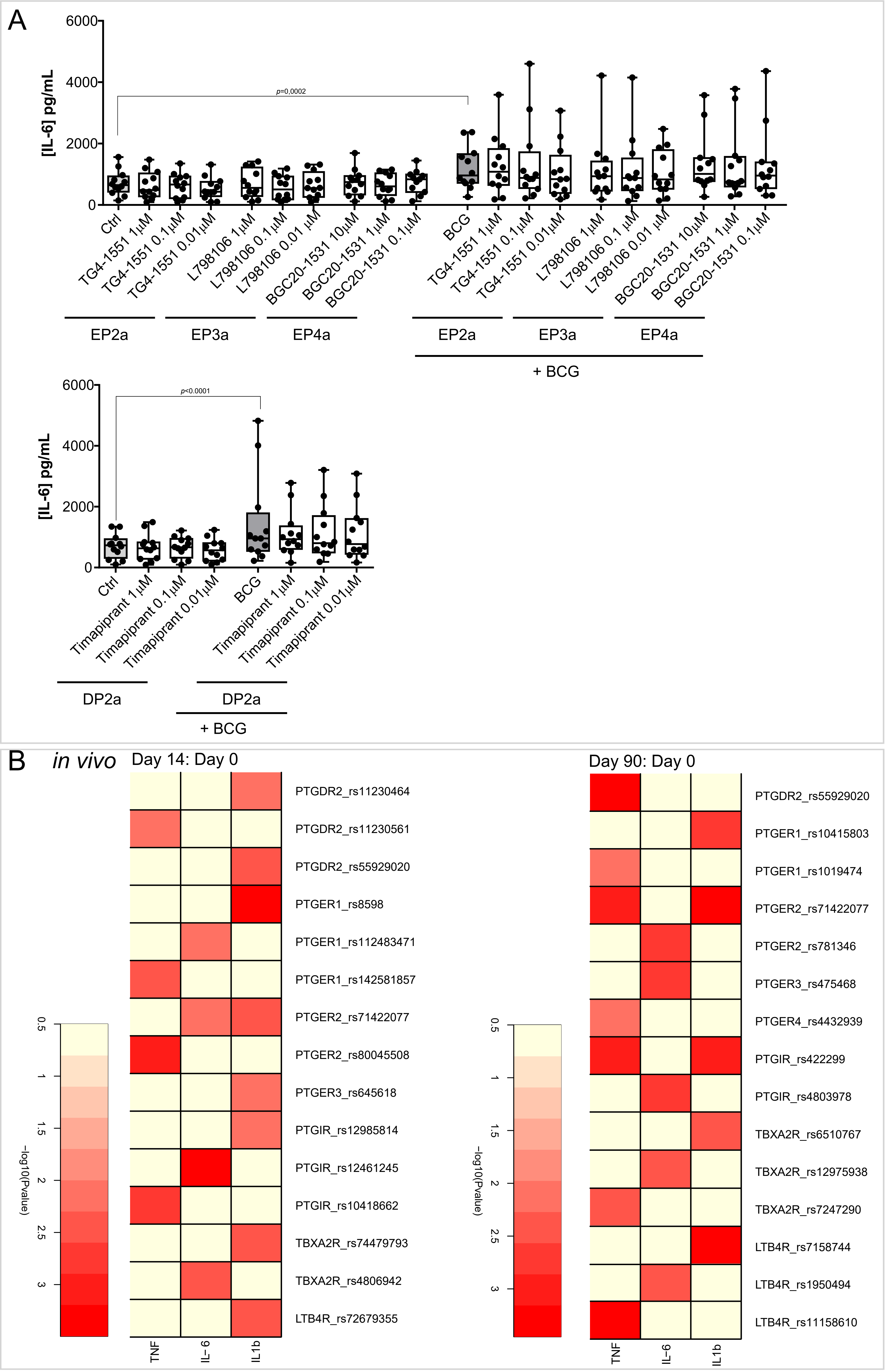

Figure S5. The role of prostanoids receptors in BCG-induced responsiveness. (A) IL-6 by BCG-trained macrophages incubated with (1, 0.1, 0.01 μM) TG4-155, (1, 0.1, 0.01 μM) L798106, (10, 1, 0,1 μM) BGC20-1531 or (1, 0.1, 0.01 μM) Timapiprant for the first 24 h and restimulatedwith LPS for another 24 h at day 6 of culture. (n=12 biologic replicates, pooled from 4 independent experiments). Mean, 10-90 percentile and whiskers extend to the most extreme points, two-way ANOVA, Sidak's multiple comparisons test. (B) Heatmaps of the p values of the association between SNPs around *PTGDR2, PTGER1, PTGER2, PTGER3, PTGER4, PTGIR, TBXA2R, LTB4R* (+/- 250kb) and the fold change of the production of TNFα, IL-6 and IL-1β upon BCG vaccination and *S. aureus ex vivo* restimulation, 14 days after vaccination (left panel) and 90 days after vaccination (right panel) (n= 278 healthy volunteers).

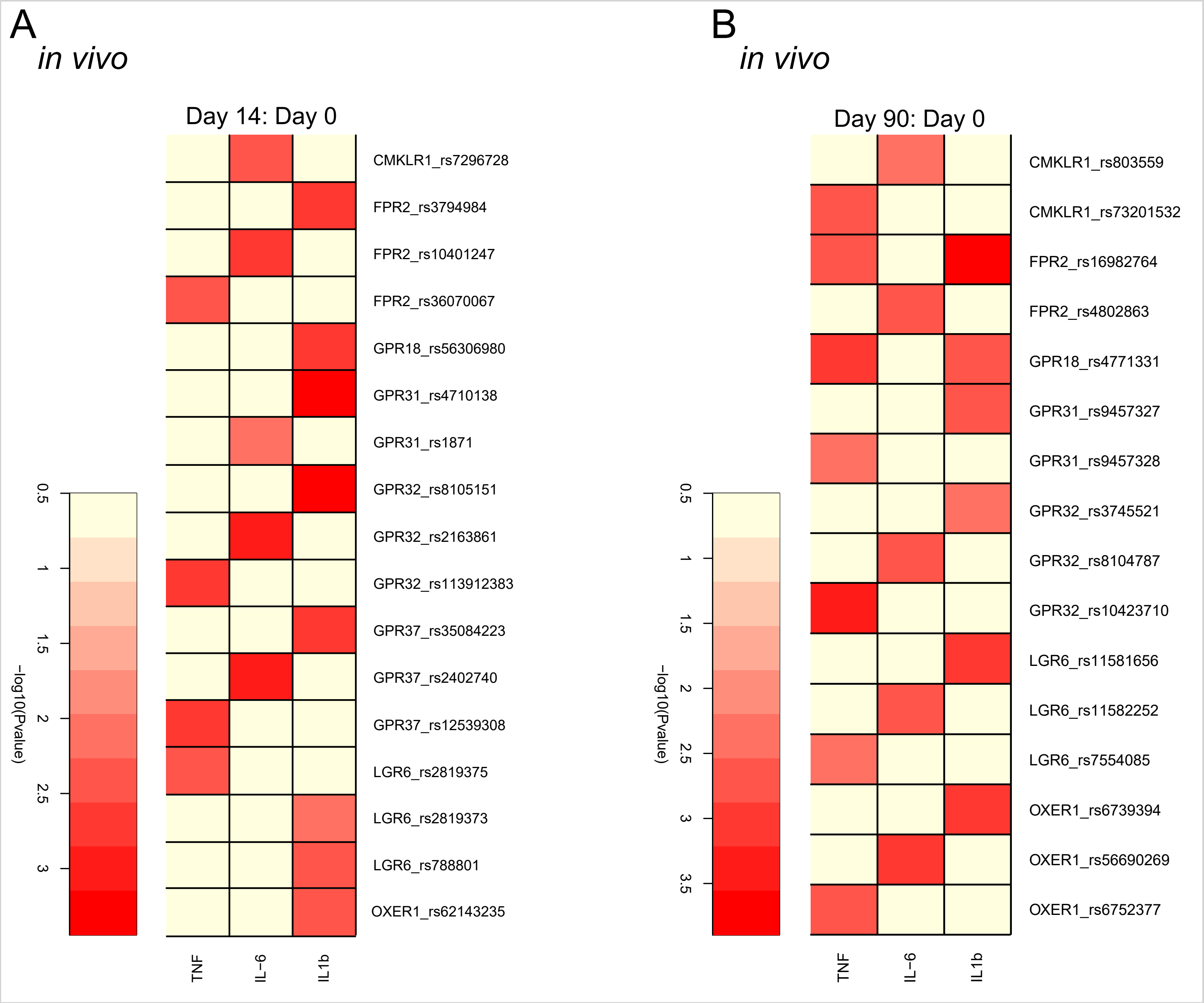

Figure S6. The role of oxylipins receptors in BCG-induced responsiveness. Heatmaps of the p values of the association between SNPs around *CMKLR1, FPR1, GPR18, GPR31, GPR32, GPR37, LGR6, OXER1* (+/- 250kb) and the fold change of the production of TNFα, IL-6 and IL-1β upon BCG vaccination and S. aureus *ex vivo* restimulation, 14 days after vaccination (left panel) and 90 days after vaccination (right panel) (n= 278 healthy volunteers).

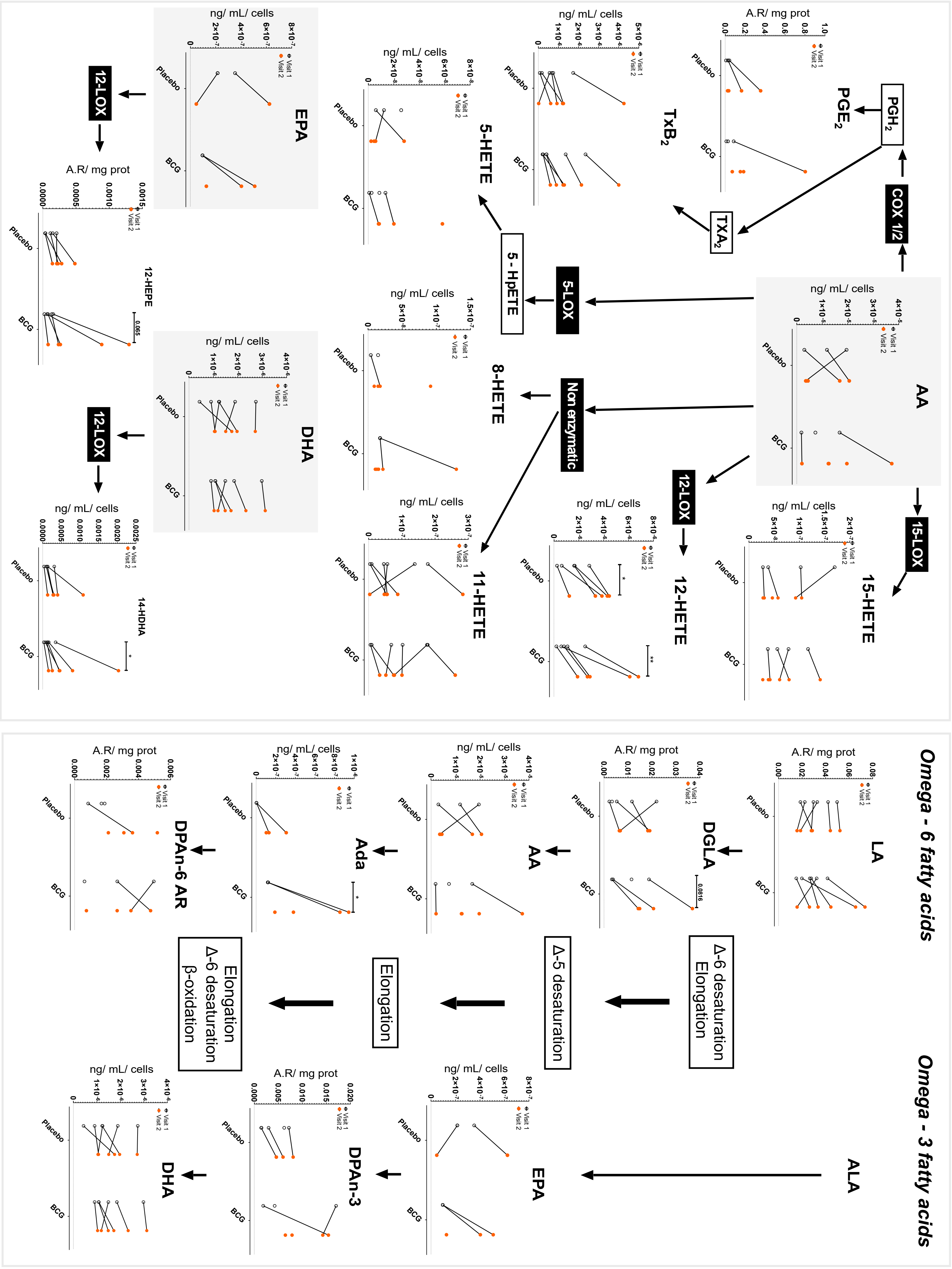

Figure S7. Concentrations (ng/mL) corrected to cell number or Area ratio (A.R) corrected to protein content of PUFA and oxylipids of *in vivo* BCG-trained response in monocytes (n=6 biological replicates), * p ≤ 0.05, ** p ≤ 0.01, two-way ANOVA, Sidak's multiple comparisons test. Data points below the technical limit of detection are not shown. One outlier per group was removed (ROUT test (Q=5%).

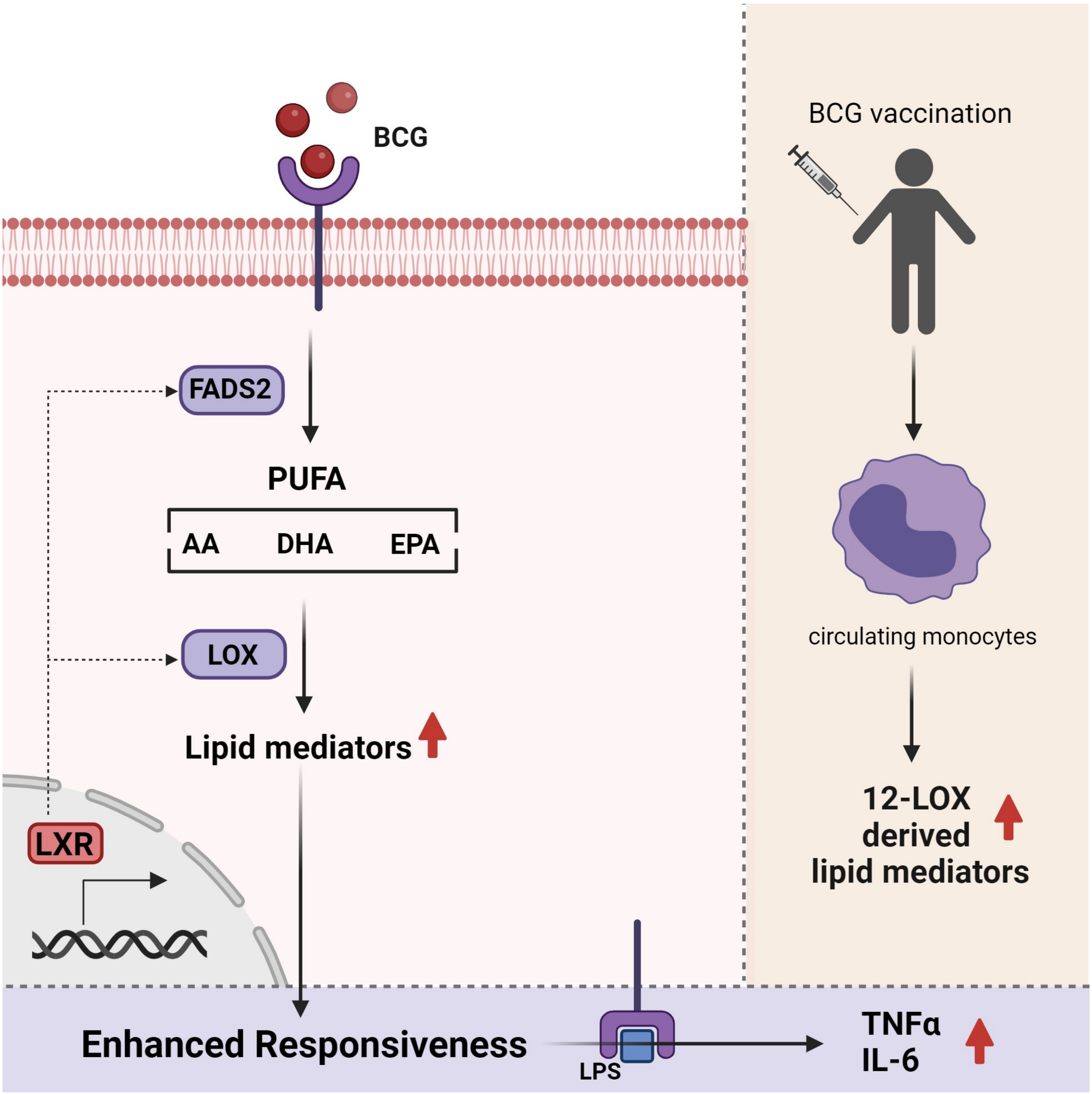

Figure S8. Graphical representation of the key findings of this manuscript. Created with BioRender.com
