# Supplementary material for: Fatty acid desaturation and lipoxygenase pathways support trained immunity
Source: Nat Commun. 2023 Nov 15;14:7385. doi: 10.1038/s41467-023-43315-x (PMC10651900; doi:10.1038/s41467-023-43315-x)
Supplement: Supplementary file 3 — Reporting Summary [file 41467_2023_43315_MOESM3_ESM.pdf]

nature portfolio

Corresponding author(s): Anaisa V Ferreira

Last updated by author(s): Oct 17, 2023

Reporting Summary

Nature Portfolio wishes to improve the reproducibility of the work that we publish. This form provides structure for consistency and transparency in reporting. For further information on Nature Portfolio policies, see our Editorial Policies and the Editorial Policy Checklist.

Statistics

For all statistical analyses, confirm that the following items are present in the figure legend, table legend, main text, or Methods section.

n/a	Confirmed	
☐	☒	The exact sample size (*n*) for each experimental group/condition, given as a discrete number and unit of measurement
☒	☐	A statement on whether measurements were taken from distinct samples or whether the same sample was measured repeatedly
☐	☒	The statistical test(s) used AND whether they are one- or two-sided<br>*Only common tests should be described solely by name; describe more complex techniques in the Methods section.*
☐	☒	A description of all covariates tested
☐	☒	A description of any assumptions or corrections, such as tests of normality and adjustment for multiple comparisons
☐	☒	A full description of the statistical parameters including central tendency (e.g. means) or other basic estimates (e.g. regression coefficient) AND variation (e.g. standard deviation) or associated estimates of uncertainty (e.g. confidence intervals)
☐	☒	For null hypothesis testing, the test statistic (e.g. *F*, *t*, *r*) with confidence intervals, effect sizes, degrees of freedom and *P* value noted<br>*Give P values as exact values whenever suitable.*
☒	☐	For Bayesian analysis, information on the choice of priors and Markov chain Monte Carlo settings
☒	☐	For hierarchical and complex designs, identification of the appropriate level for tests and full reporting of outcomes
☒	☐	Estimates of effect sizes (e.g. Cohen's *d*, Pearson's *r*), indicating how they were calculated

Our web collection on statistics for biologists contains articles on many of the points above.

Software and code

Policy information about availability of computer code

Data collection	Gen5 3.03 (Biotek, VT, USA) and StepOne v2.3 (Thermo Fisher, MA, USA) software were used to record cytokine and expression levels, respectively. Human study data was collected using Castor EDC (Amsterdam, the Netherlands). Lipid data was acquired in Analyst 1.7 (Sciex, MA, USA).
Data analysis	GraphPad prism version 5.03 and 9.01<br>Lipid data was analysed using MultiQuant 2.1 (Sciex,MA,USA)<br>Quality control of the pre- and post-imputed genetic data was performed using PLINK (v1.90b6.18 64-bit). QTL mapping was performed using the R-package Matrix-eQTL and visualization using the genetic data were also performed in R (version 3.3.2).

For manuscripts utilizing custom algorithms or software that are central to the research but not yet described in published literature, software must be made available to editors and reviewers. We strongly encourage code deposition in a community repository (e.g. GitHub). See the Nature Portfolio guidelines for submitting code & software for further information.

Data

Policy information about availability of data

All manuscripts must include a data availability statement. This statement should provide the following information, where applicable:

- Accession codes, unique identifiers, or web links for publicly available datasets
- A description of any restrictions on data availability
- For clinical datasets or third party data, please ensure that the statement adheres to our policy

The authors declare that the data supporting the findings of this study are available within the manuscript and its supplementary information files

Research involving human participants, their data, or biological material

Policy information about studies with human participants or human data. See also policy information about sex, gender (identity/presentation), and sexual orientation and race, ethnicity and racism.

Reporting on sex and gender	Genetic analysis performed using the 300BCG cohort used a linear regression model with age and sex as covariates.
Reporting on race, ethnicity, or other socially relevant groupings	Genetic analysis was performed using the 300BCG cohort, composed of of healthy individuals of Western European descent from the Human Functional Genomics Project. This cohort consists of 325 adults from the Netherlands (44% males and 56% females, age range 18 – 71 years).<br>The monocytes used for lipid mediator analysis were derived from participants of the BCG+ cohort. The participants included in this analysis are of western European descent (50% males, 50% females, age range 20-48 years, median age 24 years).
Population characteristics	Genetic analysis was performed using the 300BCG cohort, composed of of healthy individuals of Western European descent from the Human Functional Genomics Project. This cohort consists of 325 adults from the Netherlands (44% males and 56% females, age range 18 – 71 years).<br>The monocytes used for lipid mediator analysis were derived from participants of the BCG+ cohort. The participants included in this analysis are of western European descent (50% males, 50% females, age range 20-48 years, median age 24 years).
Recruitment	Genetic analysis was performed using the 300BCG cohort. Exclusion criteria were use of systemic medication other than oral contraceptives or acetaminophen, use of antibiotics 3 months before inclusion, previous BCG vaccination, history of tuberculosis, any febrile illness 4 weeks before participation, any vaccination 3 months before participation, and a medical history of immunodeficiency.The QTL mapping using the 300BCG cohort was recently published (Moorlag SJCFM et al 2021. Eur J Immunol).<br>The monocytes used for lipid mediator analysis were derived from participants of the BCG+ cohort. Exclusion criteria were allergies to any of the possible interventions; known (history of) active or latent Mycobacterium tuberculosis or with another mycobacterial species; prior BCG vaccination; acute illness 2 weeks prior to the study or (suspicion of) active infection; pregnancy; chronic use of any systemic drugs other than oral contraceptives; use of NSAIDs less than 4 weeks prior to start of the study; other vaccination in the past 3 months or expected vaccination during the study period; medical history associated with immunodeficiency; active solid or non- solid malignancy within the prior two years; hypocalcemia; inability to sit upright for 30 minutes; esophagus abnormalities.<br>Participants of both cohorts were recruited via flyers and posters at the Radboud University Medical Center (Radboudumc) and the Radboud University and online advertisements aimed at people who live around Nijmegen, the Netherlands.<br>Of note, most participants were young adults of Western European descent so our results might not be reflective of all age groups and genetic backgrounds.
Ethics oversight	The 300BCG study was approved by the local ethics committee CMO region Arnhem- Nijmegen, NL58553.091.16.<br>The BCG+ study approved by the local ethics committee CMO region Arnhem- Nijmegen, NL74082.091.20.

Note that full information on the approval of the study protocol must also be provided in the manuscript.

Field-specific reporting

Please select the one below that is the best fit for your research. If you are not sure, read the appropriate sections before making your selection.

☒ Life sciences ☐ Behavioural & social sciences ☐ Ecological, evolutionary & environmental sciences

For a reference copy of the document with all sections, see nature.com/documents/nr-reporting-summary-flat.pdf

Life sciences study design

All studies must disclose on these points even when the disclosure is negative.

Sample size	No statistical methods were used to determine sample size for the in vitro assays or in vivo lipid analysis. An estimate of 4 to 12 donors were used in experiments and we assumed this sample size would be sufficient to recognize differences between stimulations, as seen in previous studies of similar readouts and effect sizes (Arts R. et al Cell Metabolism. 2016; Bekkering S. et al. Cell. 2018).<br>For the in vivo mouse experiments sample size was determined using the Power and Sample Size Calculator using the fisher exact test, with a power 80% and a type I error 0.05, with a null hypothesis that 80% of the control group and 20% of the treatment group would develop a clinically significant colitis, defined as DAI score greater than 2.

The number of individuals included in the 300BCG study (Moorlag SJCFM et al 2021. Eur J Immunol) has been chosen based on prior cohort sizes in the Functional Genomics Project (500FG, 200FG) that demonstrated that such size numbers are sufficient for identifying significant factors for immunological assays.

Data exclusions

No data was excluded for in vitro monocytes stimulation experiments and for in vivo mouse experiments: no data was excluded.
The genetic analysis excluded three samples due to medication use (of which one was identified as a genetic outlier), and one sample due to onset of type 1 diabetes during the study.
In the in vivo lipid analysis, samples below the limit of detection of the technique and statistical significant outliers were excluded

Replication

Studies were performed in different healthy donors to rule out donor effects and to check reproducibility. The number of biological replicates and number of experiments performed are indicated in the figure legends. The in vivo mouse experiment was performed twice and replicated successfully.

Randomization

For the in vivo mouse experiments, animals were randomly assigned to each treatment group by a randomization table
Participants of the BCG+ study, from which monocytes were isolated for lipid analysis, were randomized between groups (Castor EDC, Amsterdam, the Netherlands).

Blinding

For the in vivo mouse experiments randomization was performed by an individual who did not participate in the following procedures of the experiment.
The BCG+ study was single blinded since researchers were not aware of the allocated groups during sample preparation.
For in vitro experiments, treatment groups were not blinded since each in vitro experiment was conducted by one researcher.

Reporting for specific materials, systems and methods

We require information from authors about some types of materials, experimental systems and methods used in many studies. Here, indicate whether each material, system or method listed is relevant to your study. If you are not sure if a list item applies to your research, read the appropriate section before selecting a response.

Materials & experimental systems

n/a	Involved in the study
☒	☐ Antibodies
☒	☐ Eukaryotic cell lines
☒	☐ Palaeontology and archaeology
☐	☒ Animals and other organisms
☒	☐ Clinical data
☒	☐ Dual use research of concern
☒	☐ Plants

Methods

n/a	Involved in the study
☒	☐ ChIP-seq
☒	☐ Flow cytometry
☒	☐ MRI-based neuroimaging

Animals and other research organisms

Policy information about studies involving animals; ARRIVE guidelines recommended for reporting animal research, and Sex and Gender in Research

Laboratory animals

30 C57BL/6JOlaHsd male mice (Hellenic Pasteur Institute) that were 8-10 weeks old were used. Animals were housed in groups of 5 animals per enriched type-II cage on a 12 h light-dark diurnal cycle with room temperature between 21 and 23 °C. Water and food were provided ad libitum.

Wild animals

The study did not involve wild animals.

Reporting on sex

We included only male mice as it has been shown that male mice develop a more severe form of experimentally induced colitis (Bábíčková J. et al. Inflammation. 2015)

Field-collected samples

The study did not involve samples collected from the field.

Ethics oversight

Animal experiments were conducted in the Unit of Animals for Medical and Scientific Purposes of the University General Hospital “Attikon” (Athens, Greece) according to EU Directive 2010/63/EU for animal experiments and to the Greek law 2015/2001, which incorporates the Convention for the Protection of Vertebrate Animals used for Experimental and Other Scientific Purposes of the Council of Europe (code of the facility EL25BIO014, approval number 1853/2015). All experiments were licensed from the Greek veterinary directorate under the protocol number 338087/27-05-2020.

Note that full information on the approval of the study protocol must also be provided in the manuscript.
